# The Adipocyte-Derived Hormone Leptin Has Proliferative Actions on Androgen-Resistant Prostate Cancer Cells Linking Obesity to Advanced Stages of Prostate Cancer

**DOI:** 10.1155/2012/280386

**Published:** 2012-05-28

**Authors:** M. Raschid Hoda, Gerit Theil, Nasreldin Mohammed, Kersten Fischer, Paolo Fornara

**Affiliations:** Clinic for Urology and Kidney Transplantation Centre, University Medical School of Halle/Wittenberg, Ernst-Grube-Strasse 40, 06120 Halle, Saale, Germany

## Abstract

*Background*. Because obesity may be a risk factor for prostate cancer, we investigated proliferative effects of adipocytes-derived hormone leptin on human prostate cancer cells and assessed the role of mitogen-activated protein kinase (MAPK) signaling pathway in mediating these actions. *Material and Methods*. Three human prostate cancer cell lines were treated with increasing doses of recombinant leptin. Cell growth was measured under serum-free conditions using a spectrophotometric assay. Further, Western blotting was applied to detect the phosphorylation of an ERK1/2, and a specific inhibitor of MAPK (PD98059; 40 **μ**M) was used. *Results*. In both androgen-resistant cell lines DU145 and PC-3, cell growth was dose-dependently increased by leptin after 24 hrs and 48 hrs of incubation, whereas leptin's proliferative effects on androgen-sensitive cell line LNCaP was less pronounced. Further, leptin caused dose-dependent ERK1/2 phosphorylation in both androgen-resistant cell lines, and pretreatment of these cells with PD98059 inhibited these responses. *Conclusions*. Leptin may be a potential link between obesity and risk of progression of prostate cancer. Thus, studies on leptin and obesity association to prostate cancer should differentiate patients according to androgen sensitivity.

## 1. Introduction

Among other serious diseases, obesity is known to be associated with an increased risk for a number of different cancers such as breast cancer, esophageal cancer, colon cancer, renal cell cancer, and pancreatic cancer [1, 2]. Studies using body fat measurement and disease stratification according to prostate cancer (PCa) stage have found a strong association between obesity and PCa [3–5]. In Western population with high fat intake and prevalence of obesity, the incidence of clinically significant prostate cancer and disease-specific mortality rates are increasing [3]. Meanwhile, increased levels of endogenous hormones associated with overweight and obesity, such as sex steroids, insulin, insulin-like growth factor I, and leptin, have been described as potential mechanisms linking obesity to prostate cancer [1]. In particular, the fat hormone leptin has been shown to be positively associated with prostate cancer [6–9]. Recently, there has been an increasing interest on the study of cellular and molecular mechanism of cancer by energy restriction models [10]. For instance, Berrigan et al. and Mai et al. observed that the relationship between energetic balance and cancer development could be explained to a great extent by caloric restriction, as mediated through leptin [11, 12]. The discovery and isolation of the human *obese*-gene and its protein product, leptin, have led to numerous subsequent studies linking leptin to obesity. Leptin, a 16 kDa, 167-amino acid peptide, is released mainly by adipocytes, which contributes to the concept of adipocyte and adipose tissue as an endocrine cell and an endocrine organ, respectively. After binding to its receptors in the hypothalamus, leptin induces a complex response partly resulting in regulation of body weight and energy expenditure [13, 14]. In humans, levels of leptin in the blood seem to correlate directly with body mass and may increase from levels of 1–3 ng/mL in non-obese subjects to as high as 100 ng/mL in obese individuals [15].

Several recent studies have indicated that leptin acts as a mitogenic factor in a variety of cell types including vascular endothelial and smooth muscle cells, as well as normal and neoplastic colonic, ovarian, and breast cells [16, 17]. Leptin receptors have been shown to exist on various cancer cells [18, 19]. The leptin receptor, ObR, is a member of the class I cytokine-receptor family. While the short isoforms of the leptin receptor seem to mediate the transport and degradation of leptin, the long isoform, ObRb, expressed by the hypothalamus and many peripheral cells, is reported to be responsible for most of the central and peripheral actions of leptin [19]. Both short and long isoforms of the leptin receptor have been found in prostate cell membranes [18, 20]. These receptors have been shown to be functional in terms of their ability to activate mitogenic signaling pathways. However, the exact mechanism by which leptin exerts proliferative effects on cancer cells, including those from the prostate, is not fully understood. There is emerging evidence that chronic hyperleptinemia associated with obesity might constitute a risk factor for prostate cancer [21]. We previously have shown that leptin *in vitro* is able to induce mitogenic actions in a group of human prostate cancer cell lines [22]. In the present study, we tested the hypothesis, whether the proliferative effect of leptin in prostate cancer cells is linked to the stage of androgen-sensitivity in these cells. For this purpose, we sought to evaluated any difference in response to leptin in androgen-sensitive and insensitive prostate cancer cell lines.

## 2. Materials and Methods

### 2.1. Reagents

Human recombinant leptin was purchased from Sigma Inc. (St. Louis, MO). Blocking reagent for Western blotting (skim milk), rainbow-colored protein molecular weight markers, ECL Plus (Amersham Pharmacia Biotech, Piscataway, NJ), phospho-p44/42 MAPK antibody (ERK1/2, rabbit polyclonal IgG1; Cell Signaling Technology Inc., Beverly, MA), and MEK inhibitor PD98059 (Cell Signaling Technology Inc. Beverly, MA) were obtained from the sources indicated.

### 2.2. Cell Cultures

One human androgen-sensitive human prostate adenocarcinoma cell line (LNCaP) and two androgen-resistant human prostate cancer cell lines (DU145 and PC-3) were used for these experiments (CLS Corp., Germany). PC-3 cells were cultured in Ham's F12K medium supplemented with 2 mM L-glutamine adjusted to contain 1.5 g/L sodium bicarbonate and 10% fetal bovine serum. DU145 cells were cultured in Minimum essential medium Eagle with 2 mM L-glutamine and Earle's BSS adjusted to contain 1.5 g/L sodium bicarbonate, 0.1 mM nonessential amino acids, and 1.0 mM sodium pyruvate, 90%; fetal bovine serum, 10%. LNCaP cells were cultured in RPMI 1640 media (Invitrogen, USA) supplemented with 2 mM L-glutamine adjusted to contain 1.5 g/L sodium bicarbonate, 4.5 g/L glucose, 10 mM HEPES, 1.0 mM sodium pyruvate, and 10% fetal bovine serum. Passages were carried out with trypsin-EDTA. All cell lines were grown at 37°C in an atmosphere of 95% O_2_/5% CO_2_.

### 2.3. Treatments

For all experimental procedures, cells were transferred to serum-free medium 24 hours after seeding. After a further 24 hours, leptin was added at various concentrations (5–100 ng/mL) and for different treatment times (up to 48 hours). For Western blot studies, we used only one uniform time point of 1 hour for leptin treatment (5–100 ng/mL). Control cells were treated with vehicle (PBS). Where inhibitor was used, cells were pretreated for 3 hours with PD98059 (40 *μ*M) prior to leptin addition.

### 2.4. Measurement of Cell Proliferation

The XTT colorimetric assay (Roche, Mannheim, Germany) was used to detect cell proliferation after 24 and 48 hours of incubation in the presence of leptin or vehicle. Cells were plated in 96-well plates at a concentration of 5 × 10^3^ cells per well. At designated time points, XTT, a tetrazolium salt, was added to the each well at a final concentration of 0.3 mg/mL. Plates were incubated in the presence of XTT dye for 4 h to allow for the formation of the orange formazan dye product by metabolically active cells. Absorbance was read spectrophotometrically at 450 nm using an ELISA plate reader. The data are reported as a percentage of the untreated control. Assays were performed at least five times and samples were run in triplicate.

### 2.5. Immunoblotting

Approximately 10^6^ cells were plated into each well of 6-well plates for these studies and treated with leptin for various times. All incubations were stopped by washing (×3) with ice-cold phosphate-buffered saline (PBS). Ice-cold lysis buffer was added (consisting of 1% Triton-X-100, 1 mM NaVO_4_, 1 *μ*g/mL leupeptin, 1 mg/mL pepstatin, 1 mg/mL antipain, 1 mM NaF, 1 mM EDTA, and 100 mg/mL PMSF in PBS), and the cells were incubated at 4°C for 30 minutes. Cells were then scraped into microcentrifuge tubes, centrifuged at 10,000 rpm for 10 minutes, and the supernatant retained. Aliquots of each sample were assayed to determine protein content, and samples were adjusted so that they contained equal amounts of protein. Samples were then mixed with gel loading buffer (50 mM Tris, pH 6.8, 2% SDS, 100 mM DTT, 0.2% bromphenol blue, 20% glycerol). The samples were boiled for 5 minutes and proteins separated by SDS-PAGE. Separated proteins were transferred onto a PVDF membrane (DuPont NEN, Boston, MA). The membrane was washed in 1% blocking buffer for 30 min, followed by incubation with an appropriate dilution of primary antibody against pERK1/2 or pAkt in blocking buffer for 60 minutes. This was followed by washing (×3) in Tris-buffered saline with 1% Tween (TBST). Following washes, an HRP-conjugated secondary antibody was added to the membrane in 1% blocking buffer and allowed to incubate for an additional 30 minutes. This was followed by further washing (×3) in TBST. Immunoreactive proteins were detected using an enhanced chemiluminescence detection kit (ECL, Amersham Biosciences, Piscataway, NJ) and exposure of the membrane to X-ray film. Quantification of protein phosphorylation was determined by densitometry using NIH Image software.

### 2.6. Statistical Analysis

Data are presented as means (s.e.m.). Statistical analysis was performed using SigmaPlot software v8.0 (SPSS Inc., Chicago, IL). One-way analysis of variance or, where appropriate, repeated measures ANOVA, with a Student Newman Keuls post hoc test were performed on all data. A *P* value < 0.05 was considered to be statistically significant.

## 3. Results

**Figure 1:**
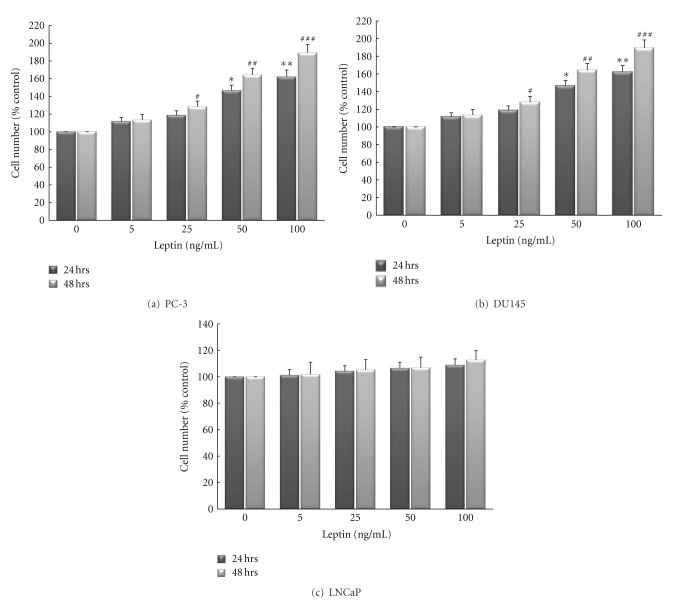
Leptin increases cell proliferation in androgen-resistant prostate cancer cell lines ((a); PC-3, (b); DU145) in a dose-dependent manner. Conversely, leptin's proliferative effect on androgen-sensitive cell line ((c); LNCaP) was much less pronounced. Cells were cultured in serum-free media for 24 hours (white bars) or 48 hours (black bars) in the presence or absence of leptin (0–100 ng/mL) and cell numbers were determined by a colorimetric XTT assay. Assays were performed at least five times and samples were run in triplicate. The data (means ± SEM) are reported as a percentage of results in untreated controls and asterisks or pound signs denote values significantly different from these cells at 24 and 48 hrs, respectively, (* or ^#^ *P* < 0.05; ** or ^##^ *P* < 0.01; *** or ^###^ *P* < 0.001 by ANOVA).

Leptin increased cell numbers in both androgen-resistant cell lines after 24 hrs and 48 hrs of incubation; whereas leptin's proliferative effect on androgen-sensitive cells was much less pronounced. As shown in Figures 1(a)–1(c), cell numbers were dose-dependently (5–100 ng/mL) increased at 24 and 48 hours after leptin treatment in DU145 and PC-3 cell lines when compared to cell numbers in serum-free control cultures. Maximal growth responses were observed after 48 h at a leptin concentration of 100 ng/mL: 161.2 ± 5.1% of control in DU145 cells (*P* < 0.001) and 182.7 ± 7.9% of control in PC-3 cells (*P* < 0.001). However, treatment of LNCaP cells with leptin (100 ng/mL) for up to 48 hours triggered only a small effect on cell proliferation (percent of control; 112.3 ± 6.1%; 100 ng/mL leptin; 48 hrs).

**Figure 2:**
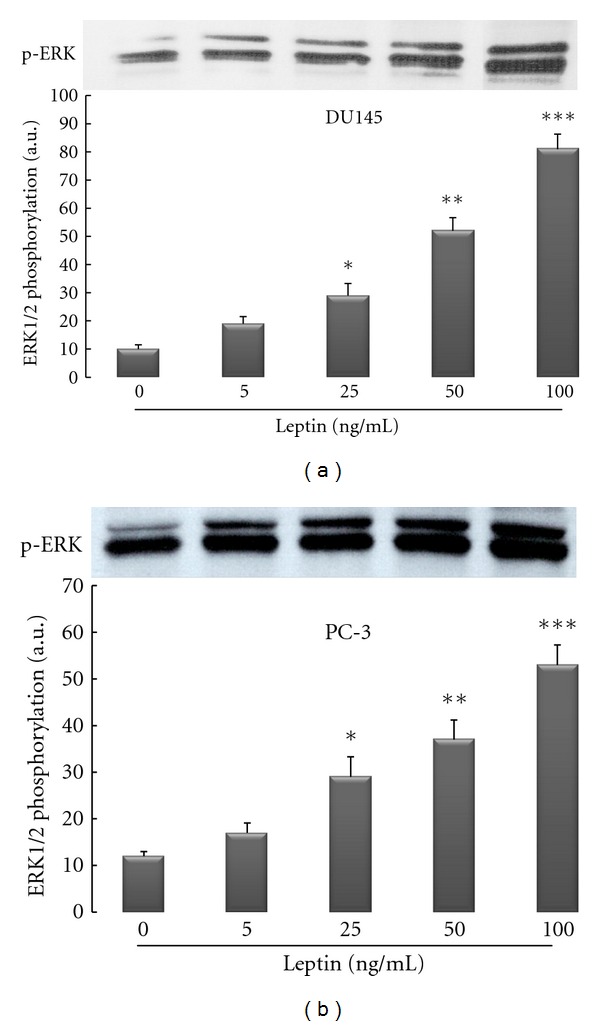
Leptin activates ERK1/2 isoforms of MAPK signaling pathway in a dose-dependent manner in androgen-resistant prostate cancer cell lines (DU145 and PC-3). Three different human prostate cancer cell lines were cultured in serum-free media for 24 hrs followed by exposure to recombinant human leptin for 1 hour. Cellular extracts were fractioned onto 12% SDS-Page and Western immunoblotting performed with a rabbit polyclonal anti-phospho-p44/42 MAPK as described in Section 2. The findings are from a single experiment representative of at least 3 similar experiments.

A common intracellular pathway that has been shown to be recruited by leptin receptors is the mitogen-activated protein kinase (MAPK) cascade [17, 23, 24]. To evaluate if leptin's mitogenic action in androgen-resistant prostate cancer cells is linked to proliferative signaling through activation of the MAPK signaling pathway, DU145, PC-3, and LNCaP cells were treated with leptin and phosphorylation of p42/44 MAPK (ERK1/2), a downstream component of MAPK pathway was measured by Western blotting using phosphospecific antibodies. As would be expected from proliferation assay data, leptin treatment evoked ERK phosphorylation in both androgen-resistant cell lines in a dose-dependent manner (Figures 2(a) and 2(b)). In LNCaP cells, however, leptin also evoked activation of ERK phosphorylation, but to a comparably lesser extent (data not shown).

**Figure 3:**
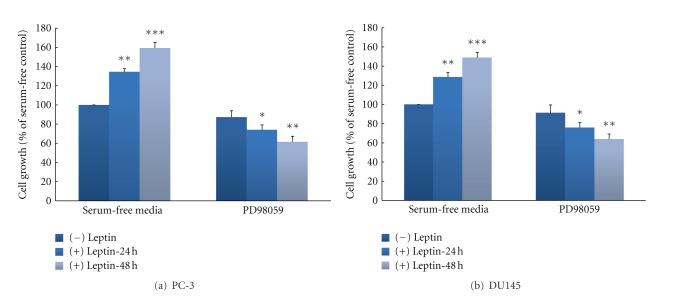
Inhibitor of MAPK attenuates leptin-induced prostate cancer cell growth in androgen-resistant prostate cancer cell lines. Cells ((a); PC-3, (b); DU145) were cultured in serum-free media for 24 and 48 hours with or without leptin (100 ng/mL). Before adding leptin, cells were pretreated with the MEK inhibitor PD98059 (PD; 40 *μ*M). Cell number was determined by the colorimetric XTT assay. Assays were performed at least five times and samples were run in triplicate. The data (means ± SEM) are reported as percentage of the untreated control and asterisks denote values significantly different from vehicle-treated cells. (*P < 0.05; **P < 0.01; ***P < 0.001 by ANOVA).

To further investigate whether leptin-stimulated MAPK activation is linked to cell proliferation in androgen-resistant cells, a specific inhibitor was used to block this signaling pathway. Leptin alone increased cell proliferation in both androgen-resistant cell lines. However, pretreatment of cell lines with the MEK inhibitor PD98059 (40 *μ*M) markedly reduced cell proliferation in both cell lines (Figures 3(a) and 3(b)).

## 4. Discussion

The prevalence of obesity is increasing among the world population [1]. The comorbidities associated with obesity represent an enormous burden on health care systems [25]. It is, therefore, important to understand the relationships between obesity and a number of diseases, including cancer, and the mechanisms involved in their interaction. In a prospectively studied cohort of 900,000 U.S. adults, Calle et al. found that obese patients were more likely to die from a number of cancers, including prostate cancer [26]. Further, some links between obesity and cancer has been also strongly documented in different cancer types, for example, breast cancer and colonic cancer [27, 28]. However, studies of obesity and prostate cancer are complicated by the fact that obesity is associated not only with excess body fat, but also with altered serum levels of numerous hormones, including testosterone, estrogen, insulin, insulin-like growth factor (IGF)-1, and leptin, all which have to some degree been linked to prostate cancer [3]. For instance, mitogenic actions of leptin in certain organs in both normal and disease states have been reported and increasing epidemiological data in humans, as well as numerous *in vitro* investigation and animal studies suggest a link between leptin and cancer growth [17, 29]. The leptin receptor isoforms (ObRa and ObRc through ObRf) have been reported in a wide variety of human and rodent tissues: heart, placenta, lung, liver, muscle, kidney, pancreas, spleen, thymus, prostate, testes, ovary, small intestine, and colon [30–32]. *In vitro*, it has been shown that human prostate cancers express the leptin receptor and leptin is able to stimulate growth of some human prostate cancer cell lines [22, 33–37]. The present study was undertaken to further investigate the effect of leptin on growth of prostate cancer cells as we have tested the hypothesis whether the increase in prostate cancer growth by leptin might be different in androgen-resistant cells compared to androgen-sensitive cells. Further, the potential involvement of major mitogenic signal transduction pathways, such as MAPK pathway was evaluated by measuring the activation of its downstream component (ERK1/2) in these cell lines. To characterize the difference in proliferative effects of leptin, three different human prostate cancer cell lines were treated with leptin. Leptin treatment for up to 48 hours increased prostate cell proliferation in a dose-dependent manner in androgen-resistant cells, with a maximal proliferative effect between concentrations of 50 and 100 ng/mL (Figures 1(a) and 1(b)). This is the range of concentration that has been measured in serum of obese patients as it has been shown that circulating serum leptin levels correlate with body fat content and obese humans have as high as 100 ng/mL (average of 40 ng/mL) serum leptin levels, whereas lean individuals have 1–3 ng/mL (average 4 ng/mL) serum leptin levels [15, 20].

However, while androgen-sensitive cells showed only a slight proliferative response to leptin treatment, there was stronger response in PC3 cells, when compared to the DU145 responses (Figures 1(a)–1(c)). The reason for the latter finding might be that DU145 cells have been speculated to have much moderate metastatic potential compared to PC3 cells which might have higher metastatic potential [38]. A difference in response to leptin among the different cell lines from the same organ and/or different organs has also seen in other studies [17, 23, 29, 34, 39].

Mitogenic actions of leptin in certain organs in both normal and disease have been reported [16, 38]. Again, for prostate cancer cells, most studies reported mitogenic and antiapoptotic effect of leptin on androgen-resistant cell lines DU145 and PC-3 cells, while androgen-sensitive cell line LNCaP cells were mostly unresponsive or not tested [21, 22, 34]. As one of the few studies, Onuma et al. reported on mitogenic actions of leptin in androgen-independent cell lines PC3 and DU145, but not in androgen-dependent cell line LNCaP-FGC [21]. However, it is noteworthy, that in contrast to our study, they used very high supra-physiologic concentrations for the cell treatments with leptin (12.5 *μ*g/mL). Also, they used very long incubation time of 5 days, which was in clear contrast to our study [21]. However, as the number of reported studies in the literature increases, the problem of reporting conflicting results regarding the proliferative and/or (anti-)apoptotic effects of leptin on PCa cells and the involved signaling mechanisms remains. As a consequence, most studies vary widely in the dose range of leptin, time-length of leptin treatment, and the models used or the parameters tested and accordingly, also in the results obtained on the effects of leptin. For instance, Somasundar et al. reported that in DU145 cells, proliferation was significantly increased by 4 and 40 ng/mL after 72 hrs of leptin treatment, as measured by MTT assay [20]. In contrast to our study, they performed their proliferation assays only at 2 time-points (24 and 72 hrs) and using only 2 doses of leptin (4 and 40 ng/mL). Thus, some more pronounced effects of leptin in higher dose ranges (50–100 ng/mL), as this also seems to be common in obese individuals [15], might have been missed by their study. Interstingly, they also observed a different pattern of mitogenic action of leptin in PC-3 cells, as these had a maximum growth response to 4 ng/mL leptin at the 24 hrs time period [20]. Further, Somasundar et al. showed also an inhibition of serum-deprivation-induced apoptosis by leptin in dose rage of 40 ng/mL (24 hrs) in PC-3 and DU145 cells [20]. However, unlike our previously published study, the found somewhat conflicting results for low-dose leptin (4 ng/mL), as this increased apoptosis in PC-3 cells and inhibited it in DU145 cells [22]. As a matter of fact, it seems that leptin effects on apoptosis in prostate cancer cells continue to be a subject for serious debate, as also some very conflicting results are available now in the literature. For instance, Samuel-Mendelsohn et al. reported very recently their surprising finding that leptin exerted proapoptotic effects in four PCa cell lines (PC3, DU145, PC3/AR and LNCaP), as assessed by four different parameters of apoptosis [40]. In these studies, they induced apoptosis in the cells using starvation medium for 48 hrs. The proapoptotic response to leptin in this study was rapid and sensitive, being maximal already with 1 ng/mL and 6 hrs of exposure [40]. The proapoptotic effect of leptin was maintained in all cell lines for up to 24 hrs, and then mostly reduced by 72 hrs [40]. As possible explanation for these conflicting results concerning the apoptosis, in can be presumed the ability of leptin to variously affect the balance between pro- and antiapoptotic signals, depending on the cell type, dose of leptin treatment, and the pathophysiological and molecular milieu active at a given point in time [20–22, 40]. Recent studies show also that the ability of leptin to affect cell growth and apoptosis and androgen receptor expression in various human prostate cancer cell lines is likely related to the complexity of the effects mediated by its full-length receptor in activating different intracellular signalling pathways [40]. Nevertheless, the exact mechanisms of these varieties in leptins effect are still unknown and because the effects of leptin can clearly vary between different types of cancer, different cancer cells, or even at different stages of progression of a given cancer cell type, an important goal to be achieved will be the development of targeted leptin receptor agonists and antagonists aimed at a specific function of leptin and/or a specific marker in a given cancer cell type. However, the ultimate goal of such studies should be to clarify whether the obesity-related hyperleptinemia is likely a risk factor for occurrence of androgen-resistency in prostate cancer. As pointed out by Ribeiro et al., this fact should already be considered in current clinical hormonal therapy for prostate cancer, which includes the use of antiandrogens, and LHRH analogues [41]. The ultimate goal of this therapy is the blockade of androgen production with subsequent decrease in androgen serum levels. Considering the inverse relationship between serum levels of cortisol, and other steroids including androgens, a blockade of androgens will be followed by an increase in leptin expression [42, 43]. Ribeiro et al. suggest that the unbalanced serum increase in leptin and decrease in androgens may facilitate androgen-independent cell growth, while downregulating androgen-dependent cells [41]. However, more studies are needed to clarify the clinical impact of this hypothesis.

Signal transduction pathways play an important role in cancer cell proliferation, apoptosis, oncogenic transformation, and tumor progression, and these pathways involve protein kinases at multiple levels [23, 44]. Therefore, Western blot studies were conducted to evaluate the pathways related to cell proliferation that are activated upon treatment with leptin. In particular, the MAPK signaling pathway is known to be important in cancer cell survival and growth. Thus, the potential involvement of MAPK signal transduction pathway in mediating the effects of leptin in prostate cell lines was examined by measuring the phosphorylation of its downstream component ERK1/2. It could be demonstrated that leptin induces ERK1/2 activation in conjunction with increased cell proliferation in both cell lines (Figures 2(a) and 2(b)). Although we did not observe this, Somasundar et al. had reported in an earlier work that the activation of different components of signal-transduction pathways upon treatment of cell lines with leptin might be depending on cell type [20]. For instance, they observed that in DU145 prostate cancer cell line, leptin increased the PI3-K pathway by activation of p-Akt in a dose-dependent manner (0–80 ng/mL) at the 4-h time point, whereas in the same cell line, there was a weak p-ERK activity in response to leptin. In the PC-3 prostate cancer cell line, p-ERK activity was increased in the dose-dependent manner and time effects were observed to 4 h [20]. Beside the fact that they did not assessed leptin effects in androgen-sensitive cell line, their finding regarding p-ERK activity is somewhat different than our findings, as we observed strong activity of p-ERK in the dose rage of 25–100 ng/mL leptin treatment in both cell lines (Figure 2). However, as already pointed out above, as a possible explanation, we suspect that the differences in results between our studies could be due to differences in the amount of time treated with leptin, as Somasundar et al. used 2–4 hours of treatment, while we used only one uniform time point of 1 hour for leptin treatment for our Western blot studies. Further, Samuel-Mendelsohn et al. reported in a very recent paper that exposure of native PC3, DU145, and LNCaP cells to leptin caused weak to modest, but statistically significant, increase in the phosphorylation of some signaling pathways engaged in apoptosis and cell proliferation, such as JAK2, STAT3, ERK1/2, and also PI3K-Akt [40]. However, it should be noted that compared to our studies, also in their study, different treatment doses of leptin (0.1 to 10 ng/mL) and very short time of exposure (7 minutes) were present. As a matter of fact, in contrast to all other studies published so far, in order to increase the signals induced by leptin exposure in the studied cell lines, they transiently overexpressed the LRb and JAK2 genes in the PCa cells by cotransfection with their cDNAs [20]. However, it is not uncommon to find differences in signal transduction pathways with different cell lines owing to their genetic deletions and/or mutations [20]. Nevertheless, it is important to notice that in our studies a specific biochemical blocker of the MAPK-pathway, the MEK inhibitor PD98059 (40 *μ*M), was able to abolish the mitogenic effects of leptin in both cell lines, indicating an involvement of this pathway in proliferation in both cell lines (Figure 3).

In conclusion, the present study showed that the effect of leptin on growth of prostate cancer cells is different according to the androgen sensitivity of the evaluated cells. It could be further confirmed that major mitogenic signal transduction pathways, such as MAPK pathway are involved in leptin exerting its mitogenic effects in these cell lines. Further investigation is strongly encouraged in order to investigate whether the ability of leptin to stimulate proliferation of androgen-resistant prostate cancer cells might represent a novel diagnostic/prognostic factor by serial measurements of the serum leptin levels during the course of a certain therapy. Additionally, activation of mitogenic signal transduction pathways by leptin could potentially serve as a target for future therapies of advanced stages of PCa.

## References

[1] Carroll KK (1998). Obesity as a risk factor for certain types of cancer. *Lipids*.

[2] Bray GA (2002). The underlying basis for obesity: relationship to cancer. *Journal of Nutrition*.

[3] Freeland SJ, Aronson WJ (2004). Examining the relationship between obesity and prostate cancer. *Reviews in Urology*.

[4] Andersson SO, Wolk A, Bergström R (1997). Body size and prostate cancer: a 20-year follow-up study among 135 006 Swedish construction workers. *Journal of the National Cancer Institute*.

[5] Mistry T, Digby JE, Chen J, Desai KM, Randeva HS (2006). The regulation of adiponectin receptors in human prostate cancer cell lines. *Biochemical and Biophysical Research Communications*.

[6] Freedland SJ (2005). Obesity and prostate cancer: a growing problem. *Clinical Cancer Research*.

[7] Baillargeon J, Platz EA, Rose DP (2006). Obesity, adipokines, and prostate cancer in a prospective population-based study. *Cancer Epidemiology Biomarkers and Prevention*.

[8] Freedland SJ, Sokoll LJ, Platz EA (2005). Association between serum adiponectin, and pathological stage and grade in men undergoing radical prostatectomy. *Journal of Urology*.

[9] Frankenberry KA, Somasundar P, McFadden DW, Vona-Davis LC (2004). Leptin induces cell migration and the expression of growth factors in human prostate cancer cells. *American Journal of Surgery*.

[10] Zhu Z, Jiang W, Thompson HJ (2002). An experimental paradigm for studying the cellular and molecular mechanisms of cancer inhibition by energy restriction. *Molecular Carcinogenesis*.

[11] Berrigan D, Perkins SN, Haines DC, Hursting SD (2002). Adult-onset calorie restriction and fasting delay spontaneous tumorigenesis in p53-deficient mice. *Carcinogenesis*.

[12] Mai V, Colbert LH, Berrigan D (2003). Calorie restriction and diet composition modulate spontaneous intestinal tumorigenesis in ApcMin mice through different mechanisms. *Cancer Research*.

[13] Friedman JM, Halaas JL (1998). Leptin and the regulation of body weight in mammals. *Nature*.

[14] Ahlma RS, Prabakaran D, Mantzoros C (1996). Role of leptin in the neuroendocrine response to fasting. *Nature*.

[15] Blum WF, Englaro P, Attanasio AM, Kiess W, Rascher W (1998). Human and clinical perspectives on leptin. *Proceedings of the Nutrition Society*.

[16] Hardwick JCH, Van Den Brink GR, Offerhaus GJ, Van Deventer JH, Peppelenbosch MP (2001). Leptin is a growth factor for colonic epithelial cells. *Gastroenterology*.

[17] Hoda MR, Keely SJ, Bertelsen LS, Junger WG, Dharmasena D, Barrett KE (2007). Leptin acts as a mitogenic and antiapoptotic factor for colonic cancer cells. *British Journal of Surgery*.

[18] Choi JH, Park SH, Leung CK, Choi KC (2005). Expression of leptin receptors and potential effects of leptin on the cell growth and activation of mitogen-activated protein kinases in ovarian cancer cells. *Journal of Clinical Endocrinology and Metabolism*.

[19] Laud K, Gourdou I, Pessemesse L, Peyrat JP, Djiane J (2002). Identification of leptin receptors in human breast cancer: functional activity in the T47-D breast cancer cell line. *Molecular and Cellular Endocrinology*.

[20] Somasundar P, Frankenberry KA, Skinner H (2004). Prostate cancer cell proliferation is influenced by leptin. *Journal of Surgical Research*.

[21] Onuma M, Bub JD, Rummel TL, Iwamoto Y (2003). Prostate cancer cell-adipocyte interaction: leptin mediates androgen-independent prostate cancer cell proliferation through c-Jun NH 2-terminal kinase. *Journal of Biological Chemistry*.

[22] Hoda MR, Popken G (2008). Mitogenic and anti-apoptotic actions of adipocyte-derived hormone leptin in prostate cancer cells. *BJU International*.

[23] Hegyi K, Fülöp K, Kovács K, Tóth S, Falus A (2004). Leptin-induced signal transduction pathways. *Cell Biology International*.

[24] Banks AS, Davis SM, Bates SH, Myers MG (2000). Activation of downstream signals by the long form of the leptin receptor. *Journal of Biological Chemistry*.

[25] James WP (2008). WHO recognition of the global obesity epidemic. *International Journal of Obesity*.

[26] Calle EE, Rodriguez C, Walker-Thurmond K, Thun MJ (2003). Overweight, obesity, and mortality from cancer in a prospectively studied cohort of U.S. Adults. *The New England Journal of Medicine*.

[27] Rose DP, Gilhooly EM, Nixon DW (2002). Adverse effects of obesity on breast cancer prognosis, and the biological actions of leptin. *International Journal of Oncology*.

[28] Liu Z, Uesaka T, Watanabe H, Kato N (2001). High fat diet enhances colonic cell proliferation and carcinogenesis in rats by elevating serum leptin. *International Journal of Oncology*.

[29] Somasundar P, McFadden DW, Hileman SM, Vona-Davis L (2004). Leptin is a growth factor in cancer. *Journal of Surgical Research*.

[30] Fei H, Okano HJ, Li C (1997). Anatomic localization of alternatively spliced leptin receptors (Ob-R) in mouse brain and other tissues. *Proceedings of the National Academy of Sciences of the United States of America*.

[31] Malendowicz W, Rucinski M, Belloni AS, Ziolkowska A, Nussdorfer GG, Kwias Z (2006). Real-time PCR analysis of leptin and leptin receptor expression in the rat prostate, and effects of leptin on prostatic acid phosphatase release. *International Journal of Molecular Medicine*.

[32] Tartaglia LA, Dembski M, Weng X (1995). Identification and expression cloning of a leptin receptor, OB-R. *Cell*.

[33] Malendowicz W, Rucinski M, Macchi C (2006). Leptin and leptin receptors in the prostate and seminal vesicles of the adult rat. *International Journal of Molecular Medicine*.

[34] Somasundar P, Yu AK, Vona-Davis L, McFadden DW (2003). Differential effects of leptin on cancer in vitro. *Journal of Surgical Research*.

[35] Stattin P, Söderberg S, Hallmans G (2001). Leptin is associated with increased prostate cancer risk: a nested case-referent study. *Journal of Clinical Endocrinology and Metabolism*.

[36] Gade-Andavolu R, Cone LA, Shu S, Morrow A, Kowshik B, Andavolu MVS (2006). Molecular interactions of leptin and prostate cancer. *Cancer Journal*.

[37] Kote-Jarai Z, Singh R, Durocher F (2003). Association between leptin receptor gene polyrnorphisms and early-onset prostate cancer. *BJU International*.

[38] Pulukuri SM, Gondi CS, Lakka SS (2005). RNA interference-directed knockdown of urokinase plasminogen activator and urokinase plasminogen activator receptor inhibits prostate cancer cell invasion, survival, and tumorigenicity in vivo. *Journal of Biological Chemistry*.

[39] Attoub S, Noe V, Pirola L (2000). Leptin promotes invasiveness of kidney and colonic epithelial cells via phosphoinositide 3-kinase-, Rho-, and Rac-dependent signaling pathways. *The FASEB Journal*.

[40] Samuel-Mendelsohn S, Inbar M, Weiss-Messer E, Niv-Spector L, Gertler A, Barkey RJ (2011). Leptin signaling and apoptotic effects in human prostate cancer cell lines. *Prostate*.

[41] Ribeiro R, Lopes C, Medeiros R (2006). The link between obesity and prostate cancer: the leptin pathway and therapeutic perspectives. *Prostate Cancer and Prostatic Diseases*.

[42] Hoda MR, El-Achkar H, Schmitz E, Scheffold T, Vetter HO, De Simone R (2006). Systemic stress hormone response in patients undergoing open heart surgery with or without cardiopulmonary bypass. *Annals of Thoracic Surgery*.

[43] Ribeiro R, Lopes C, Medeiros R (2004). Leptin and prostate: implications for cancer prevention—overview of genetics and molecular interactions. *European Journal of Cancer Prevention*.

[44] Krasilnikov M, Ivanov VN, Dong J, Ronai Z (2003). ERK and PI3K negatively regulate STAT-transcriptional activities in human melanoma cells: implications towards sensitization to apoptosis. *Oncogene*.

